# Talking to the Brain: Using Large Language Models as Proxies to Model Brain Semantic Features

**DOI:** 10.1002/hbm.70588

**Published:** 2026-07-06

**Authors:** Xin Liu, Ziyue Zhang, Jingxin Nie

**Affiliations:** ^1^ Philosophy and Social Science Laboratory of Reading and Development in Children and Adolescents (South China Normal University), Ministry of Education Center for Studies of Psychological Application, South China Normal University Guangzhou China; ^2^ Center for Studies of Psychological Application South China Normal University Guangzhou China; ^3^ Key Laboratory of Brain, Cognition and Education Sciences (South China Normal University), Ministry of Education Guangzhou China; ^4^ School of Psychology South China Normal University Guangzhou China; ^5^ Guangdong Key Laboratory of Mental Health and Cognitive Science South China Normal University Guangzhou China

## Abstract

Utilizing naturalistic stimuli while maintaining experimental rigor presents a significant challenge in neuroimaging research, primarily due to the complexities of annotation and high‐dimensional analysis. To address this, we introduce a novel paradigm that leverages multimodal large language models (LLMs) as high‐throughput, automated annotation tools to extract human‐understandable semantic features. This “talking to the brain” approach employs a Visual Question Answering (VQA) strategy to transform complex visual scenes into structured semantic feature vectors, which are then used to predict voxel‐wise brain activity. As a critical methodological validation, our model successfully replicated established neural correlates for categories such as face and building. Expanding beyond these benchmarks, we mapped the cortical activation patterns of 80 diverse semantic labels and constructed a data‐driven brain semantic similarity space. This space revealed robust clusters reflecting both functional categories and real‐world contextual associations. This innovative methodology offers a scalable solution for investigating brain semantic organization, overcoming the limitations of manual annotation and paving the way for more ecologically valid explorations of human cognition.

## Graphical abstract

We introduce a novel paradigm using LLMs to map the brain's semantic space with greater ecological validity. This provides a robust framework for integrating naturalistic fMRI data and reveals a hierarchically organized semantic similarity space across the cerebral cortex.

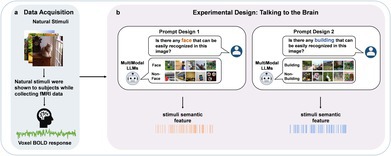

## 1 Introduction

In visual neuroscience, a central challenge lies in balancing experimental control with ecological validity. Traditional experiments often rely on artificial, isolated stimuli to test specific hypotheses, ensuring rigorous control but potentially limiting the generalizability of findings to real‐world contexts (Ioannidis 2005; Baker 2015; Open Science Collaboration 2015; Foulsham et al. 2011).

To address this, there has been a growing shift towards using naturalistic stimuli (Haxby et al. 2020; Nishimoto et al. 2011; Wen et al. 2018), such as dynamic movies or complex scenes (Bartels and Zeki 2005; Hasson et al. 2010; van der Meer et al. 2020), which elicit stronger and more reliable neural responses and provide richer insights into how the brain processes information in daily life (Sonkusare et al. 2019; Connolly et al. 2012).

However, the richness of naturalistic stimuli introduces a new bottleneck: the high‐dimensional and unstructured nature of the data makes it difficult to model specific semantic features quantitatively. Traditionally, analyzing such data required extensive manual annotation, a process that is not only labor‐intensive and time‐consuming but also prone to inconsistency when scaling to large datasets. This “annotation gap” often forces researchers to limit their analysis to a few pre‐defined categories, failing to capture the broad spectrum of semantic information present in natural scenes.

Recent advancements in multimodal large language models (LLMs) offer a powerful solution to this challenge (OpenAI et al. 2024; Gemini Team et al. 2023; Tan et al. 2024). With their exceptional capabilities in image‐text understanding and visual question answering (VQA), models like BLIP and Gemini can effectively function as high‐throughput, automated annotators (Binz and Schulz 2023; Demszky et al. 2023). Unlike human raters, these models can rapidly process thousands of images and provide consistent judgments based on explicit linguistic queries, enabling the decomposition of complex visual streams into structured semantic features (Meng et al. 2022; Wei et al. 2021).

**FIGURE 1:**
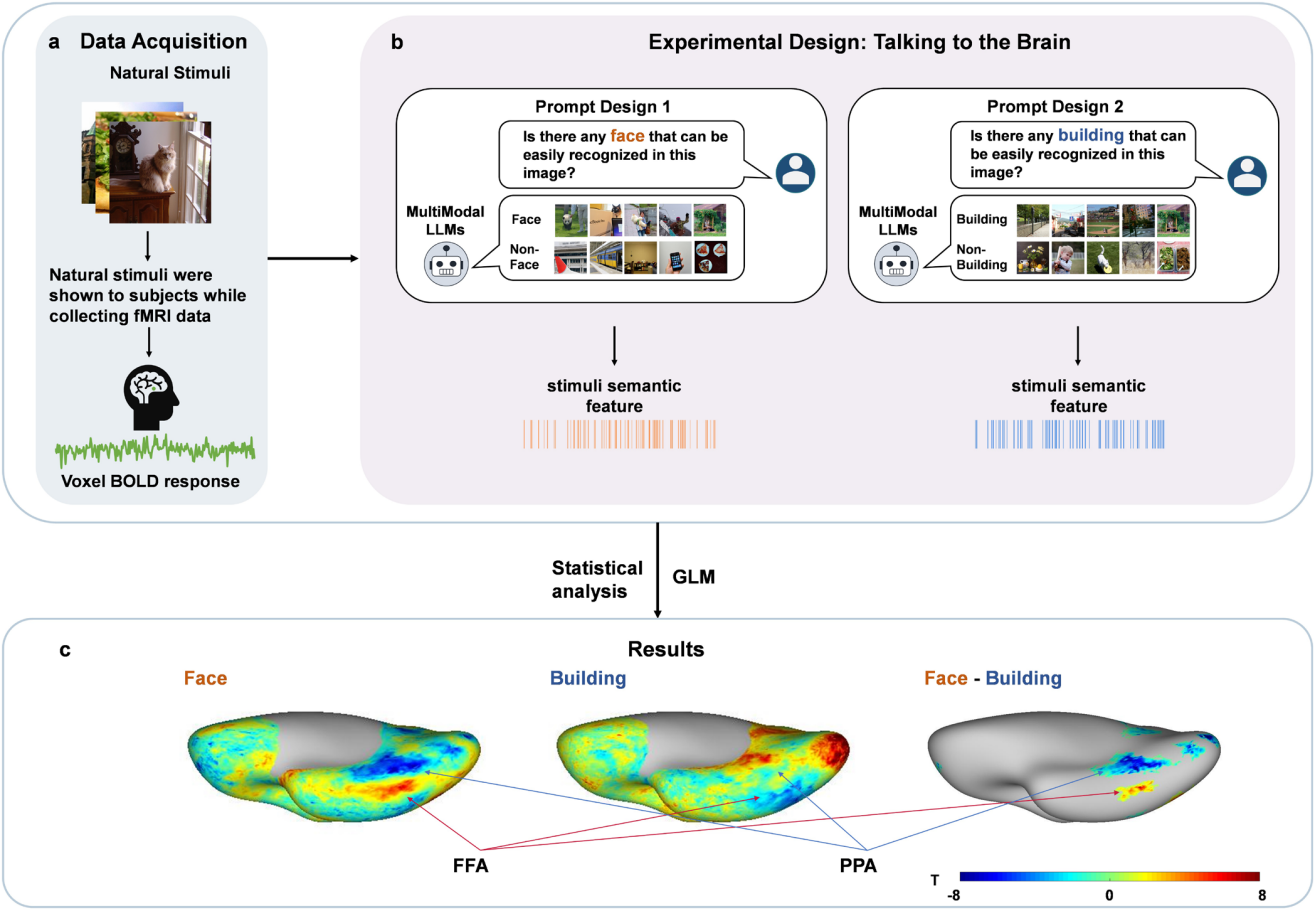
Using a large language model to extract semantic feature from natural stimuli for fMRI brain activation mapping. (a) Data acquisition. Subjects viewed natural stimuli for several hours while fMRI BOLD responses were recorded. (b) Experimental paradigm. Different prompts were designed based on the task requirements to query the large language model. The model then extracted the corresponding semantic features of the images and provided binary “yes” or “no” responses, forming the image's semantic features. A generalized linear model was applied to each cortical voxel, using the semantic features derived from the large language model to estimate brain activation patterns. The resulting activation maps for each semantic label were generated across the cerebral cortex. (c) Activation maps (ventral view, right hemisphere) of “Face”, “Building” and “Face‐Building”, respectively. The color bar represents the t‐values derived from voxel‐based modeling.

To address the limitations of traditional annotation methods, we present a novel framework that utilizes LLMs as automated tools, harnessing their advanced image‐text processing capabilities to map the semantic content of naturalistic stimuli (Antol et al. 2015; Ren et al. 2015; Zhang et al. 2016) (as shown in Figure 1). In this “talking to the brain” paradigm, we do not assume the LLM functions like a human brain; rather, we utilize the LLM to detect the presence of specific semantic concepts defined by linguistic prompts. By generating binary responses to questions, we construct interpretable feature vectors for widespread semantic categories, which are then used to predict fMRI brain activity via encoding models.

The objectives of this study are twofold. First, we aim to validate the feasibility of this LLM‐assisted approach by testing whether it can replicate well‐established neural correlates for robust categories, such as face and building (Halgren et al. 1999; Kanwisher et al. 1997; McCarthy et al. 1997; Epstein and Kanwisher 1998; Aguirre et al. 1998). Second, leveraging the scalability of our method, we extend the analysis to a broader set of 80 semantic categories to explore how these concepts are spatially organized across the cortex. This study seeks to demonstrate that LLM‐driven annotation provides an efficient, scalable pathway to decoding the complex semantic space of the human brain under naturalistic conditions.

## 2 Results

### 2.1 Validation With Well‐Established Semantic Brain Activation Pattern

As a critical sanity check to establish the reliability of our automated annotation pipeline, we first sought to replicate the neural correlates of face and building—two of the most robust landmarks in visual neuroscience. We argue that the successful identification of the FFA and PPA is not merely a replication of known facts, but a necessary methodological validation; once the framework is proven reliable against these established results, it can be logically and confidently extended to explore the cortical representations of a broader, less‐mapped semantic space.

To validate the effectiveness of using LLMs as automated annotators, we first tested whether the extracted semantic features could replicate well‐established findings in visual neuroscience. We focused on two robust categories: face and building, which are known to elicit distinct responses in specialized brain regions. Using the binary feature vectors derived from BLIP's VQA responses, we modeled the voxel‐wise activation patterns for these categories. As shown in Figure 1, images containing faces triggered significant activation in the fusiform face area (FFA), a region consistently involved in face processing (Kanwisher et al. 1997). Similarly, images of buildings caused significant activation in the parahippocampal place area (PPA), a region involved in processing environmental scenes and buildings (Epstein and Kanwisher 1998). A direct contrast between these two categories revealed distinct and opposing cortical distributions, aligning with classic domain‐specific theories of the ventral visual stream. These results confirm that our LLM‐driven framework accurately captures the fundamental link between specific visual categories and their corresponding neural substrates, providing a solid foundation for scaling the analysis to a broader semantic space.

### 2.2 Brain Activation Patterns of Different Semantic Labels

Having validated the framework, we extended the analysis to 80 semantic categories to explore their distributed representations across the cortex (Figure S1).

Consistent with the known role of the ventral visual stream in object recognition, we observed widespread activation across most labels within the lateral occipito‐temporal cortex, including the superior temporal sulcus (STS), middle temporal gyrus (MTG), superior temporal gyrus (STG), superior and transverse occipital sulci, anterior occipital sulcus, supramarginal gyrus (SMG), and the intraparietal sulcus (IPS). However, more specific patterns emerged when examining specific semantic categories. For instance, we observed a distinction between dynamic and static semantic categories at the temporo‐occipital junction (TPOJ): dynamic, outdoor sport‐related labels (e.g., sports ball, skateboard) elicited significantly positive activation compared to static semantic labels.

**FIGURE 2:**
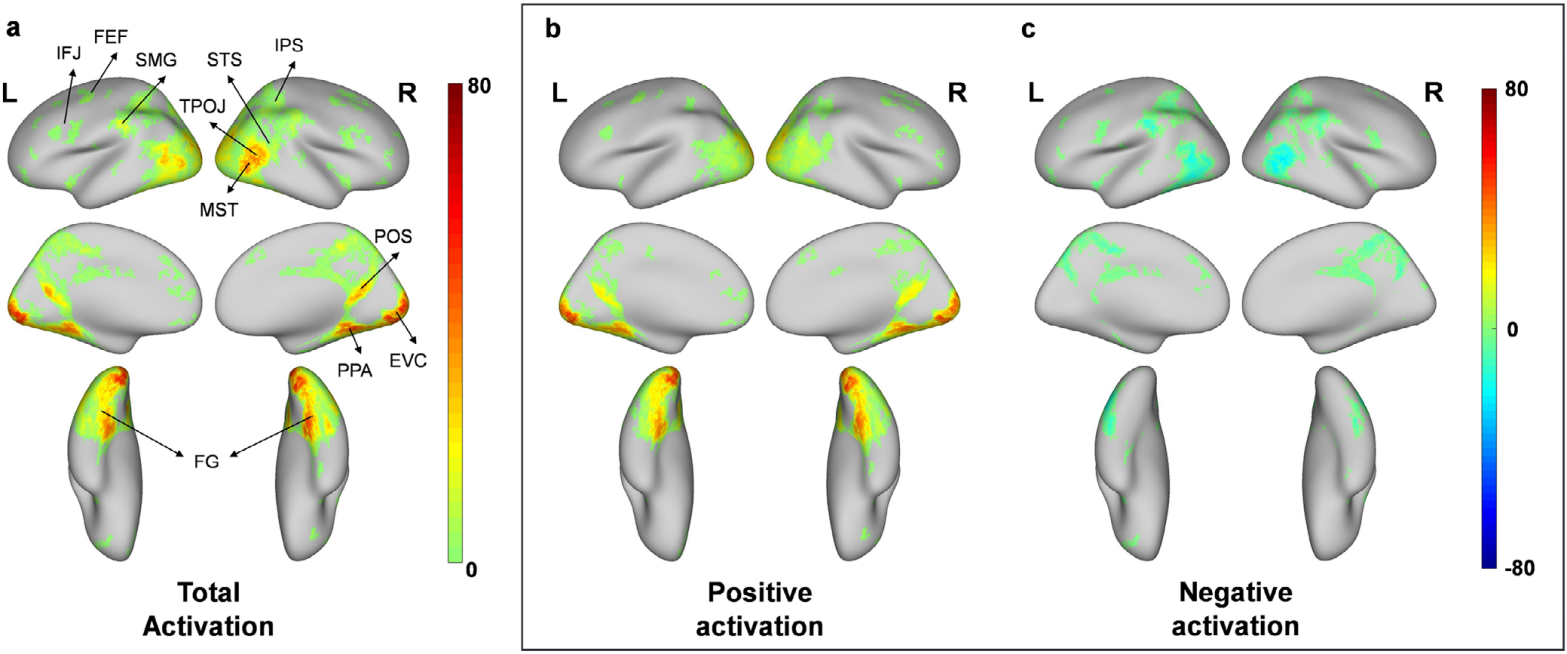
Cortical activation patterns of 80 semantic labels: overlay of total, positive, and negative activations. (a) Overlayed activation maps of the 80 semantic labels. The color bar indicates the number of semantic labels activated at each voxel. (b) Overlayed positive activation maps of the 80 semantic labels. (c) Overlayed negative activation maps of the 80 semantic labels. The color bar indicates the number of semantic labels that are positively or negatively activated at each voxel.

To identify brain regions consistently engaged across a broad range of semantic processing, we superimposed the 80 individual activation maps (Figure 2). Figure 2a demonstrates that most cortical regions participate in representing at least one semantic category, highlighting the widespread nature of semantic processing. Further analysis revealed stable positive activation during semantic image processing in several key regions, including the early visual cortex (EVC), parieto‐occipital sulcus (POS), fusiform gyrus (FG), STS, PPA, and frontal eye field (FEF), as shown in Figure 2b. In contrast, consistent negative activation was observed in the IPS, medial superior temporal (MST), and the TPOJ, as shown in Figure 2c. These results highlight both shared and distinct brain activation during semantic processing. While regions within the ventral visual pathway and the TPOJ, along with the EVC, showed stable activation across many semantic categories, other regions exhibited more selective responses. This analysis reveals a complex interaction of activation and deactivation across different brain regions during semantic processing, suggesting a sophisticated functional organization beyond simple category‐specific responses.

### 2.3 Brain Semantic Similarity Space

**FIGURE 3:**
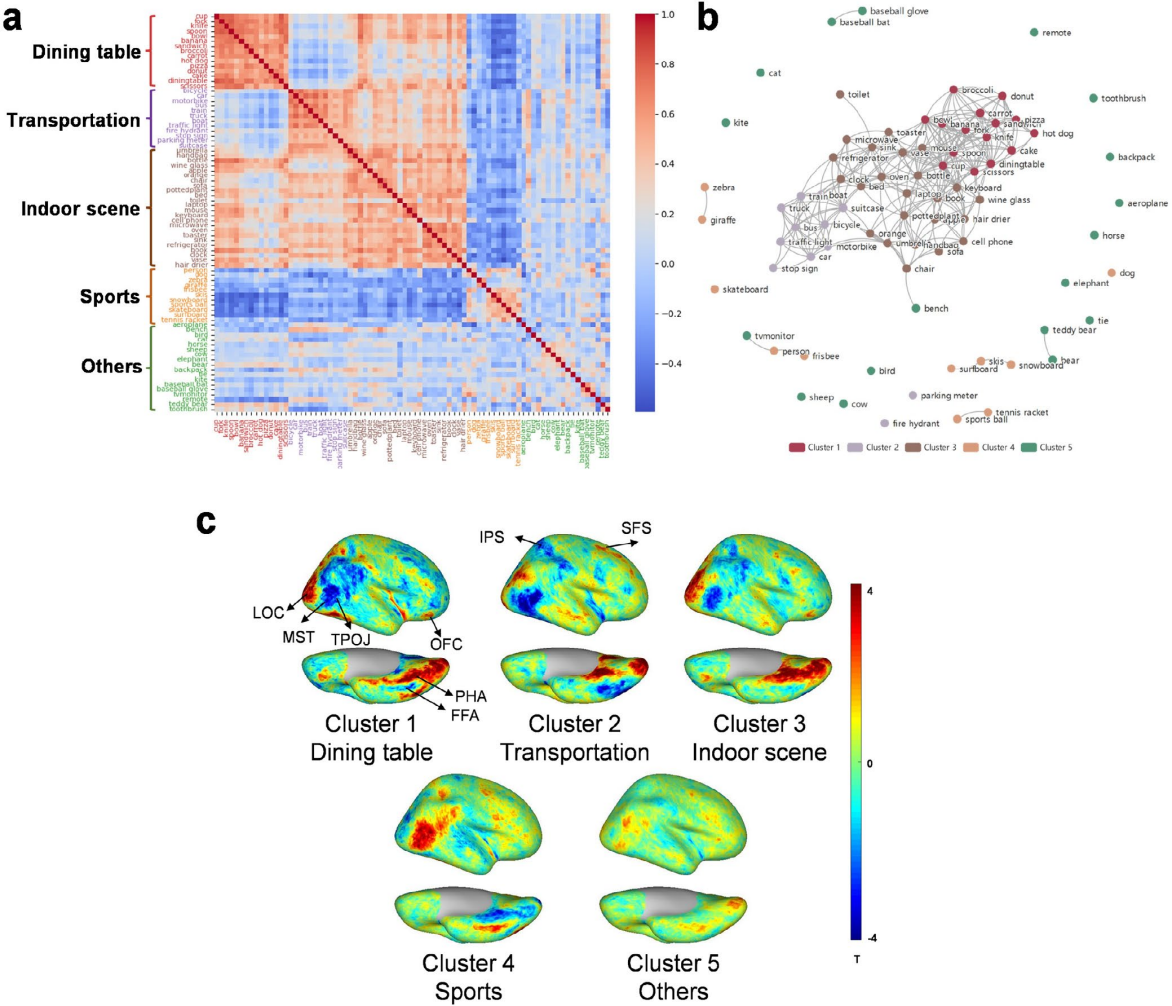
Brain semantic similarity space: Activation clusters and clustered functional relationships. (a) The similarity matrix. The correlation coefficients (color bar) reflect the relationships between activation patterns of the labels. (b) Semantic similarity space visualized at *r* = 0.55. The graph illustrates relationships between semantic labels based on pairwise correlations of their activation patterns. Nodes represent semantic categories, with colors indicating clustered groupings, and edges represent suprathreshold correlations. The semantic similarity space across additional correlation thresholds (*r* = 0.45–0.65) is provided in the Supporting Information. (c) Average whole‐brain activation maps (right hemisphere) for each of the five clusters. The color bar represents the t‐values derived from voxel‐based modeling (left brain see Figure S2).

To understand the organizational logic of these semantic features in the brain, we constructed a neural similarity matrix based on the Pearson correlations between the pairwise whole‐brain activation maps of all 80 labels. We characterized this relational architecture as a brain semantic similarity space. Agglomerative hierarchical clustering was applied to the whole‐brain activation patterns of the 80 labels, which completely data‐driven partition them into five distinct functional clusters (Figure 3a), with the optimal number of clusters determined by the silhouette coefficient. These higher‐level categories were defined post hoc based on the intrinsic functional similarity relationships clustered by the algorithm. These clusters reflect meaningful contextual associations: (1) dining table (e.g., cup, banana, pizza), (2) transportation (e.g., bicycle, bus, traffic light), (3) indoor scene (e.g., chair, bed, refrigerator), (4) sports (e.g., frisbee, skis, snowboard), and (5) others (e.g., backpack, tie, kite). The optimal number of clusters was determined by maximizing the silhouette coefficient.

As shown in the similarity matrix in Figure 3a, the labels within cluster 1, which mainly related to food and tableware, exhibit highly similar activation patterns across the cortical surface. This cluster shows a closer association with cluster 3, which includes common indoor objects such as furniture, electronics, and household appliances. Notably, items like bottle, wine glass, and apple in cluster 1 show strong correlations with labels in cluster 3. Cluster 2 consists mainly of transportation vehicles and road infrastructure, with strong correlations observed among the vehicles. In contrast, cluster 4, which includes sports equipment, shows high intra‐cluster correlation, while the label “person” and others show weaker or even negative correlations with clusters 1, 2, and 3. Cluster 5 contains a more diverse set of labels, including animals, some sports equipment, and household items; however, it shows limited intra‐cluster correlations.

The relational structure of these categories is visualized as a semantic similarity space in Figure 3b, where nodes represent semantic categories and edges denote suprathreshold similarity relationships. Clusters 1 (food) and 3 (indoor) exhibited high inter‐cluster connectivity, forming a tightly coupled core within the space, whereas clusters 4 and 5 exhibit relatively low intra‐ and inter‐cluster connectivity, appearing more peripheral in the space. To ensure the robustness of this space topology, we performed a threshold sensitivity analysis across a range of correlation coefficients (*r* = 0.45–0.65). The core community structure—such as the clustering of transportation and food items—remained stable across all tested thresholds, confirming that the similarity space visualized at *r* = 0.55 captures the essential backbone of the brain's semantic organization. The semantic similarity space at multiple correlation thresholds (*r* = 0.45, 0.50, 0.55, 0.60, and 0.65) is provided in the Supporting Information.

While these clusters represent distinct semantic categories, their activation profiles revealed both shared and distinct patterns. Clusters 1 (dining table), 2 (transportation), 3 (indoor scene), and 4 (sports) showed significant activation in the ventral visual stream and TPOJ, suggesting a common role in processing diverse semantic information. More specific differences were observed when comparing activation patterns across clusters. For example, Clusters 1 and 3 showed positive activation in the orbitofrontal cortex (OFC). The TPOJ and the MST showed significant positive activation in Cluster 4 (sports), while showing negative activation in Clusters 1, 2, and 3. The lateral occipitotemporal cortex (LOC) showed significant positive activation in Clusters 1 and 3, but a different activation pattern in Cluster 4. The IPS showed significant activation in Clusters 2, 3, and 4. Cluster 2 also exhibited significant positive activation in the SFS. The FG showed positive activation in Clusters 4 and 5, while the PPA showed negative activation in these clusters. Conversely, Clusters 1, 2, and 3 showed strong positive activation in the PPA. The activation patterns of Clusters 1 and 3 were similar, whereas Cluster 2 (transportation) showed a distinct pattern, particularly with significant negative activation in LOC.

## 3 Discussion

This study introduces a novel paradigm, “talking to the brain”, which aims to bridge the gap between complex naturalistic stimuli and neural decoding by leveraging multimodal LLMs as automated annotation tools. By using the extensive pre‐trained knowledge of LLMs, we provide a high‐throughput “language” for modeling the brain's intricate semantic features. Our findings demonstrate that LLMs can serve as effective proxies for identifying cortical activation patterns across a vast array of categories. The high consistency between our LLM‐derived maps for face and building and well‐established landmarks like the FFA and PPA validate the reliability of this framework.

A vital advantage of this LLM‐mediated approach is its ability to overcome the constraints of traditional event‐related fMRI designs. Unlike studies that isolate a few pre‐defined stimulus types (Zarahn et al. 1997). Leveraging the generalized knowledge of LLMs (OpenAI et al. 2024; Gemini Team et al. 2023; Li, Shi, et al. 2023; Radford et al. 2021; Liu et al. 2023), our paradigm allows for the post hoc extraction of features from any naturalistic image or movie frame. This flexibility enables researchers to explore the brain's response to an ecologically valid spectrum of concepts without being restricted by the experimental design used during data acquisition. While we acknowledge that LLMs categorize visual content based on statistical patterns that may not perfectly mirror human biological processing, their role as objective, automated feature extractors provides a scalable solution for annotating the “massive data” generated by naturalistic experiments.

The semantic similarity space derived from our data‐driven clustering analysis reflects the brain's functional organization of conceptual knowledge. The resulting clusters—such as food, transportation, and sports—do not merely reflect linguistic groupings but capture context‐dependent neural associations. For instance, the clustering of dynamic sports‐related items corresponds with the observed activations in regions like the MST and TPOJ, which are implicated in higher‐order motion and scene processing. By focusing on neural representational similarity rather than imposed linguistic hierarchies, we allow the brain's own activation patterns to define the relational structure of the semantic space.

A significant contribution of this framework is its potential for facilitating a more comprehensive and ecologically valid mapping of the brain's semantic space by overcoming the bottlenecks of traditional methodology. Compared to manual annotation, our LLM‐based approach offers three distinct advantages. First, our method is fully automated and highly scalable. While manual labeling is labor‐intensive and difficult to apply to the “massive data” generated by naturalistic experiments, LLMs can process these stimuli effortlessly, allowing for high‐throughput semantic mapping. Second, the use of LLMs ensures a high degree of linguistic and structural consistency across labels (Hou et al. 2024), effectively eliminating the inter‐rater variability inherent in human annotation. This consistency provides a powerful framework for integrating neuroimaging data across diverse studies and sites. By extracting standardized semantic features, researchers can combine data from multiple sources to overcome challenges such as small sample sizes, thereby enhancing statistical power and supporting more generalizable conclusions (Li et al. 2020). Finally, this paradigm offers post hoc flexibility. Unlike traditional experimental designs that require categories to be pre‐defined during data acquisition, our approach allows researchers to query and extract arbitrary semantic features after the experiment is complete. This “talking to the brain” capacity enables the exploration of neural representations for concepts that were not even initially envisioned in the study design. However, we must emphasize a precise boundary of this flexibility: this post hoc querying capacity is strictly constrained by the content ecology and visual distribution of the original stimuli. If a specific concept (e.g., “robot”) is entirely absent from the naturalistic video or image repository, the automated annotation will naturally fail to yield viable predictors. Therefore, the flexibility of our tool is not an optimization for generating unpresented information, but rather a powerful lens to re‐evaluate and flexibly decompose the hidden multi‐dimensional feature space already embedded within rich, naturalistic datasets. Moreover, the consistency of our results across different LLM architectures (BLIP and CLIP using VQA and feature correlation method, as demonstrated in Table S1) reveals the robustness and generalizability of our method. These combined strengths suggest that advanced LLMs like GPT4 (OpenAI et al. 2024) and Gemini (Team et al. 2023) will further expand the potential for decoding the complex semantic landscape of the human brain.

However, it is important to acknowledge that there are several limitations that warrant consideration of this paradigm. While the current sample size is sufficient for group‐level inference using our robust GLM framework, future studies should aim for larger cohorts to further validate these semantic maps. The fMRI data used in this study were collected during passive viewing and movie‐watching tasks, which primarily engage relatively shallow levels of semantic processing. While this approach enhances ecological validity by capturing spontaneous neural responses to natural stimuli, it may not fully tap into the deep, abstract semantic knowledge elicited by active judgment tasks. Future research should apply this LLM‐based framework to datasets involving active semantic manipulation to further explore the depth of the brain's semantic architecture. There is a notable methodological limitation lies in our downsampling strategy for dynamic movie stimuli. To align the high‐frequency continuous video with the low‐frequency discrete fMRI measurements (TR = 2000 ms), we extracted only the single middle frame within each 2‐s temporal window. While computationally efficient, this single‐frame extraction approach inherently discards substantial visual and temporal information from the remaining frames within that interval. Because the biological hemodynamic response function (HRF) is inherently slow and integrates neural activity over a temporal window of several seconds, the recorded BOLD signal inevitably encompasses the cumulative visual experience of multiple sequential frames rather than a single instantaneous snapshot. Consequently, querying a single frame may introduce noise or fail to capture highly dynamic transitions in the stimulus. A theoretically superior alternative for future iterations would be to continuously extract visual features across all video frames within each TR and aggregate them through visual semantic feature averaging or temporal pooling before predicting brain responses. Acknowledging this, our current implementation should be viewed as a baseline validation of the automated VQA pipeline, and transitioning to temporal feature‐averaging methods represents an essential direction for optimizing naturalistic encoding models.

Furthermore, we acknowledge that LLMs operate as statistical “black boxes” that lack the embodied cognition and real‐world experience of the human brain. While they provide an efficient feature space for automated annotation, the semantic features extracted by LLMs are based on text‐image co‐occurrence statistics and may not fully capture the biological depth of human semantic processing. Additionally, naturalistic stimuli inherently involve correlations between high‐level semantics and low‐level visual attributes (e.g., color, texture, and shape). While our focus was on validating the LLM as a scalable semantic tool, future work could incorporate low‐level visual models (such as Gabor filters) as covariates to further isolate abstract semantic signals from visual confounds.

Besides, while our LLM‐based framework provides a scalable solution for mapping semantic features, future research could significantly strengthen these findings by incorporating meta‐analytic decoding. A logical next step would be to benchmark the LLM‐derived activation maps against large‐scale, automated neuroimaging syntheses such as Neurosynth analysis. By performing meta‐analytic decoding, we can quantitatively assess the spatial correlation between the neural patterns identified by our VQA‐based approach and the consensus maps generated from thousands of previously published fMRI studies. For instance, using tools like the Neurosynth decoder would allow us to “reverse‐infer” whether the activation clusters we attribute to specific semantic labels (e.g., “social interaction” or “tools”) align with established functional topographies in the literature. Such a comparison would provide a rigorous, biological ground truth for our results, helping to determine the extent to which simplified binary annotations can capture the complex, higher‐order cognitive representations of the human brain. Ultimately, bridging the gap between AI‐driven automated annotation and meta‐analytic validation will be essential for establishing a more objective and standardized framework for decoding the semantic landscape of naturalistic cognition.

## 4 Method

### 4.1 Dataset

This study used data from two distinct sources, involving a total of 11 participants and 78,520 natural image stimuli.

#### 4.1.1 Natural Scenes Dataset (NSD) (Allen et al. 2022)

The NSD dataset includes data from eight healthy adult participants. Functional neuroimaging data were acquired using an ultra‐high‐field 7 T Siemens Magnetom MRI system. The T2‐weighted functional scans were collected using a gradient‐echo EPI sequence with whole‐brain coverage at a 1.8‐mm isotropic spatial resolution and a 1600‐ms temporal resolution (84 axial slices, slice thickness: 1.8 mm, slice gap: 0 mm, TR = 1600 ms, TE = 22.0 ms, flip angle = 62°, FOV = 216 × 216 mm^2^, matrix size = 120 × 120, in‐plane acceleration factor iPAT = 2, multiband slice acceleration factor = 3). During the experiment, these participants viewed approximately 73,000 color natural scene images drawn from the Microsoft COCO dataset (covering 80 common object categories), with each participant viewing approximately 10,000 unique natural scene images across 30 to 40 scanning sessions.

#### 4.1.2 Purdue Movie Dataset (Wen et al. 2018)

This dataset, obtained from the Integrated Brain Imaging Laboratory at Purdue University, consists of functional data from three healthy volunteers (female, age: 22–25, with normal vision). The imaging data were acquired using a 3 T MRI system equipped with a 16‐channel receive‐only phase‐array surface coil. The functional data were collected at a 3.5‐mm isotropic spatial resolution and a 2000‐ms temporal resolution using a single‐shot, gradient‐recalled echo‐planar imaging sequence (38 interleaved axial slices, slice thickness: 3.5 mm, in‐plane resolution: 3.5 × 3.5 mm^2^, TR = 2000 ms, TE = 35 ms, flip angle = 78°, FOV = 22 × 22 cm^2^). Participants passively watched dynamic movies featuring diverse real‐world visual content (e.g., human/animal movements, scenes), contributing 11.47 h of fMRI responses to 3.07 h of movie stimuli each. To align the continuous visual stream with the discrete fMRI measurements (TR = 2000 ms), a downsampling strategy was applied via custom Python scripts. For each 2‐s TR temporal window, the exact middle video frame (e.g., the 30th frame in a 30‐fps sequence within that 2‐s window) was extracted as the representative visual stimulus for that volume, resulting in 5520 unique natural images across the total movie duration. This high‐throughput frame extraction approach ensures that the model captures the stable visual and semantic core of each discrete scan interval.

### 4.2 Multimodal Large Language Model for Semantic Feature Extraction

The Natural Scenes Dataset (NSD) uses stimuli from the Microsoft COCO dataset, which includes 80 easily recognizable object categories (Lin et al. 2014). To extract semantic features from these images, we employed the pre‐trained multimodal large language model BLIP (Bootstrapping Language‐Image Pre‐training) (Li, Li, et al. 2023). Its pre‐training incorporates graph‐text contrastive loss, graph‐text matching loss, and language modeling loss, which together enhance its semantic comprehension capabilities, enabling strong performance in image‐text retrieval and captioning tasks.

In this study, we used BLIP to encode image semantics through a Visual Question‐Answering approach. For each semantic label we are interested in, a specific prompt was designed to query BLIP; all prompts followed a consistent grammatical structure: “Is there any [label] that can be easily recognized in this image?” (e.g., “Is there any face that can be easily recognized in this image?”). BLIP received both the image and the prompt as input, and its binary “yes” or “no” response to each prompt was then encoded to represent the semantic presence of the label. This method provides an efficient and targeted approach to extract semantic information aligned with the defined object categories.

### 4.3 Model Fitting

We employed a regression analysis to assess how well the extracted semantic features could explain participants' brain activity during visual stimulation. For each participant, the semantic features generated by the large language model (based on image stimuli and corresponding prompts) served as predictors in the regression analyses.

The semantic feature encoding produced a visual semantic time course of binary “yes” or “no” responses for each analyzed stimulus. The temporal alignment and formatting of these predictors were tailored to the distinct experimental designs of the two datasets. For the NSD dataset, because the stimuli consisted of static images presented at discrete intervals, the binary semantic vectors directly matched the categorical presentation of each fMRI volume without requiring temporal smoothing. In contrast, for the Purdue Movie dataset, the dynamic and continuous nature of the video stimuli necessitated a rigorous hemodynamic modeling pipeline to account for the slow and low‐pass properties of the blood‐oxygen‐level‐dependent (BOLD) response. Specifically, for each semantic category in the Purdue dataset, the complete, chronologically intact binary sequence derived from the movie frames was first convolved with a standard canonical hemodynamic response function (HRF, double‐gamma function with a positive peak at 4 s). This convolution procedure transformed the discrete frame‐wise annotations into continuous biological predictors while accurately preserving the temporal blurring and hemodynamic latency inherent to dynamic naturalistic viewing. Following this dataset‐specific predictor formatting, a response‐balancing procedure was executed independently for each semantic category within each dataset to mitigate statistical bias arising from severe class imbalance based on the original underlying binary distributions. We applied random undersampling to the majority class to ensure an equal feature of active and inactive instances. For the NSD data, the target static image trials and their corresponding neural volumes were sub‐sampled. For the Purdue data, the undersampling indices were determined based on the original underlying binary distributions, and both the convolved continuous predictors and their corresponding functional volumes at the discarded time points were systematically removed in tandem.

To address the inherent heterogeneity between the 7T (NSD) and 3T (Purdue) datasets, all individual fMRI data were transformed into a standardized surface space and normalized prior to model fitting. We conducted a GLM regression analysis (Friston et al. 1994) for each voxel on every participant's cortex. This analysis calculated the coefficient of determination (R^2^) and t‐value to quantify the strength and significance of the relationship between each voxel's activity and the semantic features.

Individual regression outcomes were statistically summarized and modeled using a group‐level GLM, followed by statistical testing. To control the false positive rate arising from multiple comparisons, we applied cluster correction based on Random Field Theory (RFT) (Worsley et al. 1996). Using Monte Carlo simulations (Forman et al. 1995), we determined the appropriate cluster size threshold to correct for multiple comparisons by evaluating the spatial contiguity of activation and considering the spatial correlation of voxels. By treating subjects as a random factor, this approach accounts for inter‐subject variability and potential site‐specific differences, ensuring a more robust and generalizable estimation of semantic features despite the modest sample size.

For cluster correction, we used a voxel‐level significance threshold of *p* < 0.01 with a 3 mm full‐width at half‐maximum (FWHM) smoothing kernel. Based on Monte Carlo simulations, the minimum cluster volume for significance under a two‐tailed test was determined at a cluster‐level FWE‐corrected threshold of *p* < 0.01. All data analysis and correction were performed using DPABISurf software (Yan et al. 2021).

### 4.4 Semantic Similarity Space Construction

For each semantic label, we obtained a whole‐brain activation map (t‐values from the GLM). Activation patterns for individual labels were projected onto the cerebral cortex. We then generated a semantic similarity matrix by computing the Pearson correlation between pairwise cortical activations for each label by computing pairwise Pearson correlations between these activation maps across all 80 labels, generating a similarity matrix that reflects the degree to which different semantic concepts evoke similar patterns of brain activity. This similarity matrix, which reflects the semantic relationship, was transformed into a distance matrix:

(1) distance=1–similarity.

This conversion shifted the similarity matrix range from [−1, 1] into a distance matrix range of [0, 2]. The optimal number of semantic clusters was determined by maximizing the silhouette coefficient (Rousseeuw 1987). Agglomerative hierarchical clustering (Day and Edelsbrunner 1984) was applied to the whole‐brain GLM activation maps of the 80 semantic labels to explore representational distinctions in a purely data‐driven manner. This clustering method iteratively merged the most similar clusters by minimizing intra‐cluster variance, and the 80 individual labels were subsequently assigned to 5 higher‐level categories based entirely on the mathematical cutting of the dendrogram. Finally, we constructed a cortical activation similarity graph to visually represent these clustered functional relationships. In this space, nodes represented individual semantic labels, with attributes for name and cluster membership. Edges connected labels that exhibited strong correlations, indicating potential semantic relationships. This space visually captures the relationships and clustering structure among the semantic labels. We employed a force‐directed graph to visualize the label correlation space. The connectivity is defined by a precomputed similarity matrix where an edge exists only if the correlation coefficient between two labels surpasses a predefined threshold. To avoid redundancy, the graph is treated as undirected, and duplicate links are pruned.

Furthermore, labels are organized into high‐level semantic categories (e.g., sports, transportation). Each category is assigned a unique color palette within the pyecharts (https://github.com/pyecharts/pyecharts) framework, allowing the visualization to simultaneously represent both local pairwise dependencies and global semantic groupings.

## Author Contributions

X.L. and J.N. jointly designed the analysis, interpreted the results. X.L., J.N., and Z.Z. wrote the paper. X.L. performed most of the experiments and analyses.

## Funding

This work was supported by the Research Center for Brain Cognition and Human Development (2024B0303390003), Striving for the First‐Class, Improving Weak Links and Highlighting Features (SIH) Key Discipline for Psychology in South China Normal University, and Key‐Area Research and Development Program of Guangdong Province (2019B030335001).

## Ethics Statement

The datasets analyzed in this study are publicly available (the Natural Scenes Dataset and the Purdue Movie Dataset). In the original studies, all human participants provided written informed consent, and the experimental protocols were formally approved by the respective Institutional Review Boards. Because this study involved only the secondary analysis of existing, publicly accessible, and anonymous data, it is exempt from additional institutional ethical review.

## Conflicts of Interest

The authors declare no conflicts of interest.

## Supporting information


**Figure S1:** Brain activation patterns on different semantics.
**Figure S2:** Average whole‐brain activation analysis revealed distinct patterns across the clusters (left hemisphere).
**Figure S3:** Threshold sensitivity analysis for the semantic similarity space.
**Table S1:** Comparison of experimental results across Blip and CLIP using different semantic feature extraction method.

## Data Availability

The NSD dataset (Allen et al. 2022) is publicly available on https://naturalscenesdataset.org/. Purdue Movie dataset (Wen et al. 2018) is publicly available on https://purr.purdue.edu/publications/2809/1. All analyses were performed using Python and DPABISurf (Yan et al. 2021). The fMRI data were analyzed with nilearn (https://nilearn.github.io/stable/index.html). Part of model fitting and statistical analysis was conducted using Statsmodels (Seabold and Perktold 2010).
