# Supplementary material for: Talking to the Brain: Using Large Language Models as Proxies to Model Brain Semantic Features
Source: Hum Brain Mapp. 2026 Jul 6;47(10):e70588. doi: 10.1002/hbm.70588 (PMC13334217; doi:10.1002/hbm.70588)
Supplement: Supplementary file 1 — Figure S1: Brain activation patterns on different semantics. Figure S2: Average whole‐brain activation analysis revealed distinct patterns across the clusters (left hemisphere). Figure S3: Threshold sensitivity analysis for the semantic similarity space. Table S1: Comparison of experimental results across Blip and CLIP using different semantic feature extraction method. [file HBM-47-e70588-s001.docx]

**Using Large Language Models as Proxies to Model Brain Semantic Feature**

Xin Liu, Ziyue Zhang, Jingxin Nie*

*Jingxin Nie

Email: niejingxin@gmail.com.

**This PDF file includes:**

Supplementary Text

Figs. S1 to S3

Tables S1

**Supplementary Text**

To validate the feasibility of using artificial neural networks as proxies for psychological experiments, we tested two different models to simulate semantically relevant brain activations. Blip (Bootstrapping Language-Image Pre-training) (Li et al., 2022) was tested using two methods: feature similarity and visual question answering (VQA), while the CLIP was tested through feature similarity alone. CLIP (Contrastive Language-Image Pre-Training) (Radford et al., 2021) is a multimodal model that jointly trains an image encoder (such as ResNet50 or Vision Transformer) and a text encoder (based on the Transformer architecture) to predict the correct match of image and text. CLIP excels in zero-shot learning tasks, achieving accurate predictions without the need for additional training data (Mokady et al., 2021). In our experiments, CLIP employed a feature similarity method for image representation encoding. The method inputs crafted prompts and images into a large language model, such as “This image contains a face that can be easily recognized.” The text encoder and image encoder then generate two separate eigenvector, respectively. The cosine similarity between these two eigenvectors serves as the semantic features encoding of the image. Before model fitting, we addressed potential bias from imbalanced responses by balancing the dataset for the VQA method. This step was unnecessary for feature similarity analyses, as they are not affected by response imbalances.

Using the semantic label “face” as an example, we compared the three approaches. Visualization results revealed that both Blip and CLIP achieved high consistency in activation patterns across the cerebral cortex. Notably, all three methods showed significant activation in FFA, a region consistently linked to facial recognition in previous studies, aligning well with our findings. Among the methods, VQA showed strongest performance and was selected for further experiments. Using this approach, we analyzed individual semantic labels. Consistent with previous research, the semantics of “face” and “building” were closely associated with specific brain regions. These results confirm the reliability of our experimental paradigm.

**
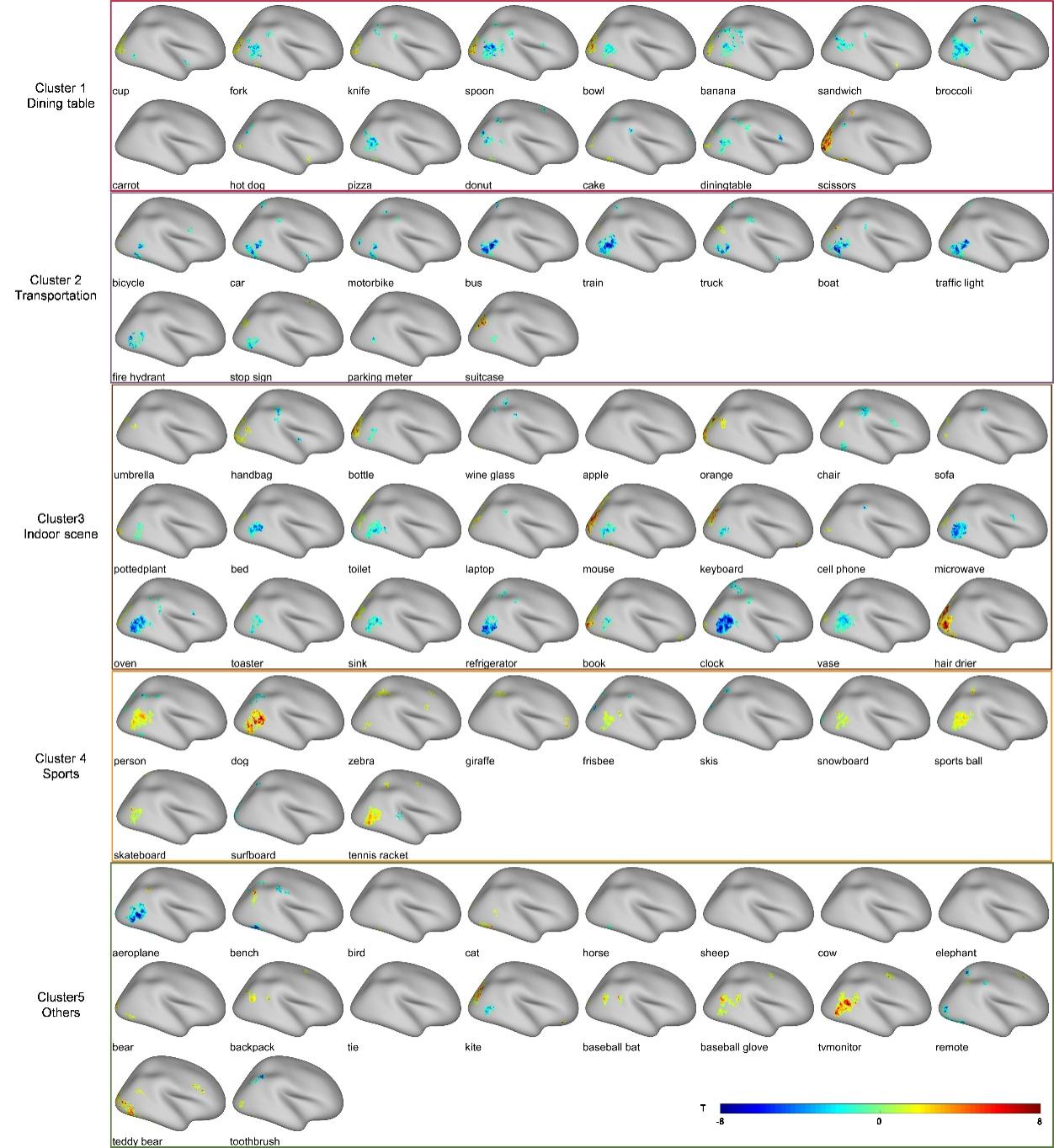
**

**Figure S1 Brain activation patterns on different semantics.**

We calculated cortical activation results for the 80 labels from COCO dataset (Lin et al., 2014), focusing on single semantic categories, The color bar represents the t-values derived from voxel-based modeling. Hierarchical clustering revealed commonalities in semantic activation patterns within each cluster. In cluster 1, which includes kitchen utensils (e.g., fork, knife) and food items (e.g., banana, sandwich), we observed consistent negative activation in the TPOJ region, while positive activation in the LOC region. Cluster 2 comprised semantic labels related to traffic scenarios. These labels showed negative activation in the TPOJ, MST regions at the temporo-occipital junction, and the IPS region in the parietal lobe. Cluster 3 contained semantic labels mainly associated with indoor scenes, including kitchen contexts (e.g., microwave, refrigerator) and electronic products (e.g., mouse, keyboard). These labels showed negative activation in the TPOJ region and positive activation in the LOC region, similar to the activation patterns observed in cluster 1. Cluster 4 included semantics related to animals (e.g., dog, zebra) and sports (e.g., frisbee, skis). Most labels in this cluster showed positive activation in the TPOJ and MST regions. Sports-related semantics and the “person” label displayed similar activation patterns, with significant activation in the STS region. Cluster 5 contained the remaining results. The activation patterns for “aeroplane” in the TPOJ and MST regions resembled those of traffic-related semantics in cluster 2. Meanwhile, the activation patterns for “baseball bat” and “baseball glove” in the TPOJ region were similarly to those of sports-related semantics in cluster 4.


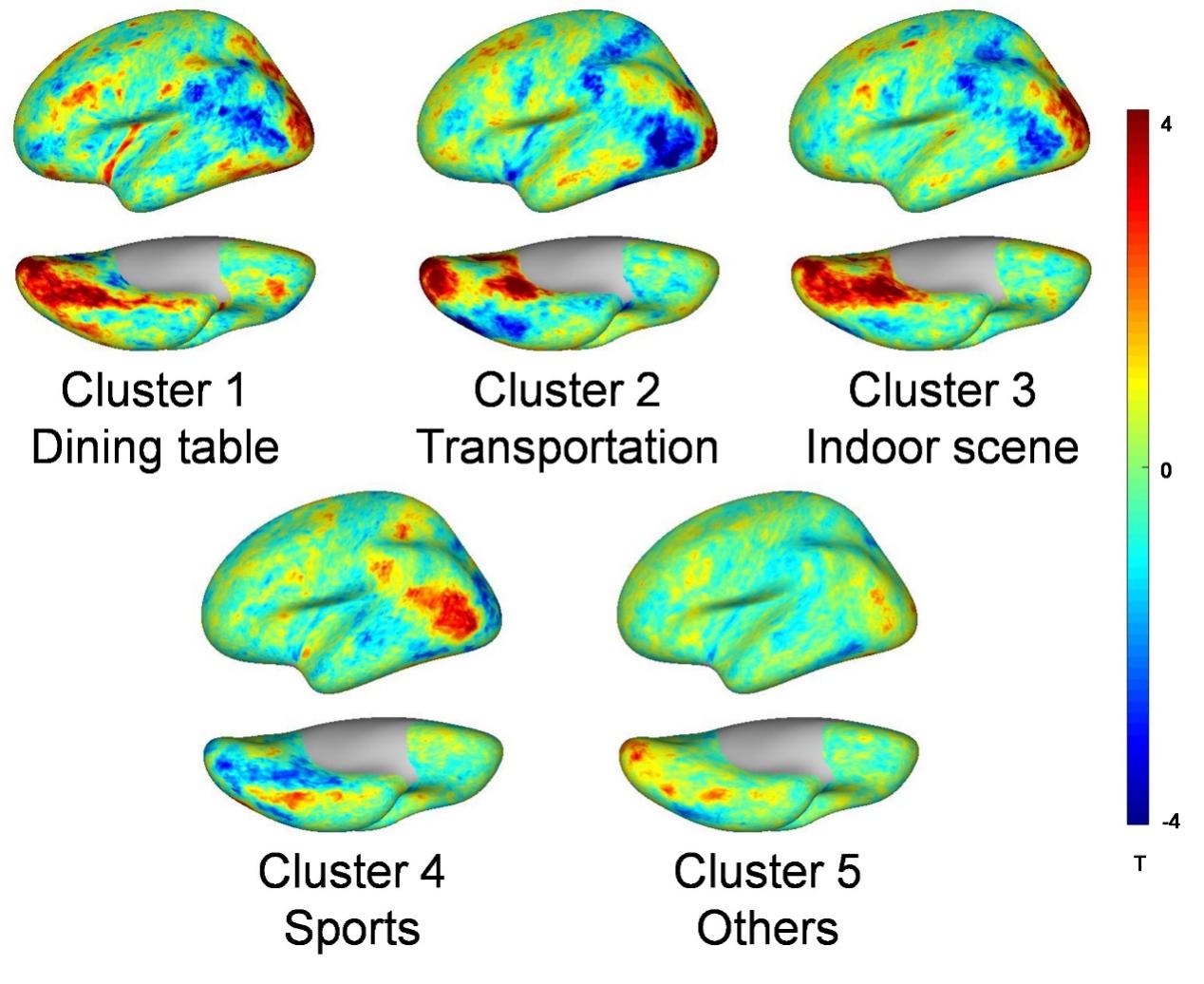


**Figure S2 Average whole-brain activation analysis revealed distinct patterns across the clusters (Left hemisphere).**

The color bar represents the t-values derived from voxel-based modeling. The activation patterns in the left hemisphere are symmetrical to that in the right hemisphere.

**
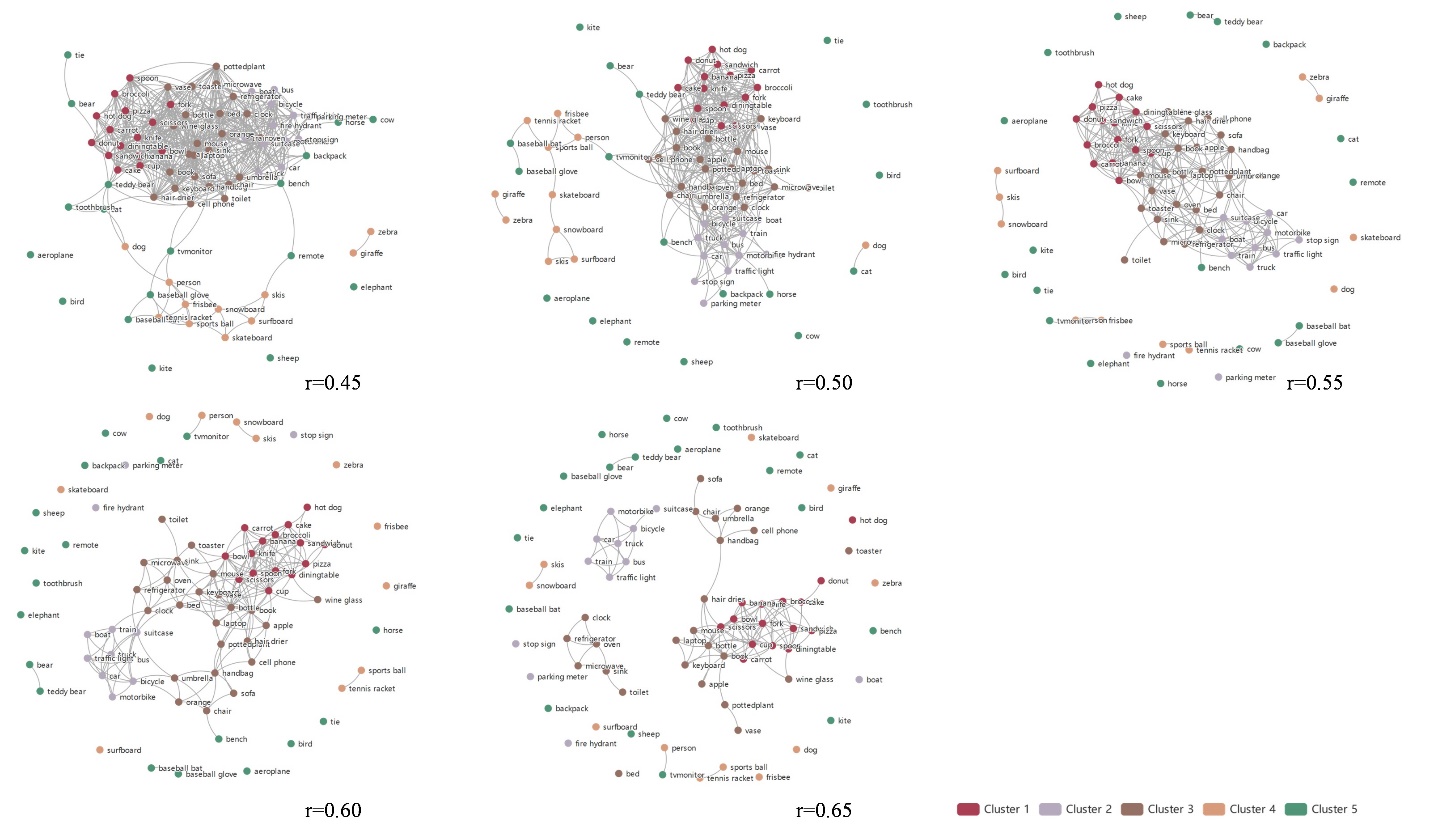
Figure S3 Threshold sensitivity analysis for the similarity space.**

The semantic similarity space (based on pairwise correlations between category-wise cortical activation maps) is shown at five different correlation thresholds: r = 0.45, 0.50, 0.55, 0.60, and 0.65. Nodes represent semantic labels, with colors indicating cluster membership (as defined in Figure 4a in the main text). Edges denote correlations above the specified threshold. The core community structure (e.g., clustering of food-related items, animals, and man-made objects) remains largely consistent across thresholds, with higher thresholds progressively removing weaker edges while preserving the overall network topology. The threshold of r = 0.55 used in the main Figure 4b was chosen as it provides a clear visualization while maintaining the key community structure.

**Table S1 Comparison of experimental results across Blip and CLIP using different semantic feature extraction method.**

| Model | Method | Mean t-value of FFA |
| --- | --- | --- |
| Blip | VQA | 2.81859 |
| Blip | Feature similarity | 2.79926 |
| CLIP | Feature similarity | 2.09704 |
